# Coupling of Modes in Step-Index Plastic Optical Fibers by Using D-Shape Technique

**DOI:** 10.3390/s24092707

**Published:** 2024-04-24

**Authors:** Cláudio Márcio F. Silva, Gefeson M. Pacheco, Jognes Panasiewicz, Luis A. Rabanal Ramirez

**Affiliations:** 1 Departamento de Micro-Ondas e Optoeletrônica, ITA—Instituto Tecnológico de Aeronáutica, São José dos Campos 12228-900, Brazil; gpacheco@ita.br; 2 Departamento de Engenharia Elétrica, UNIFOA—Centro Universitário de Volta Redonda, Volta Redonda 27240-560, Brazil; 3 Divisão de Eletrônica Espacial e Computação, INPE—Instituto Nacional de Pesquisas Espaciais, São José dos Campos 12227-010, Brazil; jognes.panasiewicz@inpe.br; 4 Departamento de Ciência da Computação, UENF—Universidade Estadual do Norte Fluminense, Campos dos Goytacazes 28013-602, Brazil; luis.ramirez@uenf.br

**Keywords:** POF, steady-state distribution (SSD), D shape

## Abstract

This article presents a technique for reducing the stabilization length of steady-state modes in step-index plastic optical fibers (POFs), which is significant for sensor networks, Internet of Things, signal processing, and data fusion in sensor systems. The results obtained with the com- putational tool developed suggest that the D-shape created in the POF effectively reduces the stabi- lization length of the modes and, by extension, minimizes the dispersion effects of the modes by filtering out high-order modes. Applying the analysis to commercial POFs, the authors experimen- tally verified a reduction in the stabilization length of modes from 27 to 10 m and from 20 m to 5 m. Reducing the mode stabilization length minimizes the bit error rate (BER) in short-length SI-POF- based optical links operating at 250 Mbp/s. A reduction from 7.6 × 10^−7^ to 3.7 × 10^−10^ was achieved.

## 1. Introduction

Plastic optical fibers (POFs) offer several advantages over glass fiber that make them viable and versatile for various applications where flexibility, cost, ease of installation, resistance to harsh environments, and short-range performance are essential considerations [1,2,3]. Step-index POFs (SI POFs), here named POFs, are widely used in several fields, ranging from home networks, fiber to the home (FTTH), Local Area Networks (LANs), the automotive industry, aviation, Unmanned Aerial Vehicles (UAVs), Internet of Things (IoT), and medical applications, among others [4,5,6]. In such systems, it is important to elevate the data transmission rate as high as possible according to the sensor numbers used and the field of application.

The development of POFs started in the decade of the 1960s but only became viable in the 1980s [1]. A POF is a multimodal optical fiber with high attenuation due to its intrinsic characteristics. A POF is mainly used in the visible spectrum region, although applications are also in the near-infrared region [1]. Poly Methyl Methacrylate (PMMA) is the most common material used in the manufacturing of POFs [7,8] and presents typical values of attenuation in the visible spectrum; for example, in the red region at 650 nm, the attenuation is 130 dB/Km, which is much more significant than 0.2 dB/Km at 1550 nm for glass optical fibers (GOFs) [3].

To achieve high data rates in POFs, one must establish mode coupling and minimize modal dispersion [7,9]. By promoting a uniform energy distribution between the modes, mode coupling minimizes the effects of modal dispersion, which is a crucial limitation of the bandwidth of multimode fibers.

In shorter lengths of fiber, where balancing the distribution of modes is more difficult to achieve naturally, mode coupling helps to stabilize the bandwidth, slowing its decline [10,11]. Mode coupling occurs when the light energy is redistributed among the different modes as it propagates through the fiber; this phenomenon helps to equalize the arrival time at the receiver of the various modes, thereby reducing modal dispersion. The coupling of modes passes through two steps. The first step is the equilibrium mode distribution (EMD), which balances the energy distribution between the different propagation modes supported by the POF. In this condition, the POF has a single attenuation value per unit length [12]. The modes interact and stabilize after the EMD but exchange energy until they reach the steady-state distribution (SSD). Upon reaching the SSD, the behavior of the modes changes regardless of the conditions of the light entering the fiber [13]. The POF reaches the EMD at a certain length, termed the Coupling Length (LC), and it attains SSD at another length, denoted as the Mode Stabilization Length (Zs). The SSD condition is ideal for achieving high transmission rates in POFs because in this condition, there is a reduction in modal dispersion and an improvement in signal integrity. The ZS must be decreased to achieve SSD in short POF segments and allow for higher data rates over shorter distances. In this work, if we consider the need for increased transmission rates in embedded systems, small and lightweight systems, laboratory instrumentation, and critical military applications (aircraft, ships, combat vehicles, and campaign communication systems), the transmission of high data rates in short POF stretches may be necessary. Considering the advantages of the POF over conventional glass fibers, this study proposes using the D shape technique, widely used in fiber sensor systems [14,15], to achieve SSD in short-length POFs. This approach’s merit is its simplicity and low cost, as it does not require additional components. This paper is divided into six sections, starting with the introduction. The coupling between the modes is then discussed in Section 2, and the components and experimental setup used to perform the measurements are shown in Section 3. The experimental results are presented in Section 4, and the bit error rate (BER) test is presented in Section 5. Lastly, the conclusions are provided in Section 6.

## 2. Modes and Coupling

Mode coupling determines the energy between the modal contents in the POF. This phenomenon gradually changes the distribution of the input energy along the length of the fiber and consequently significantly impacts the transmission characteristics of the fiber [1,16]. Mode coupling in optical fibers is primarily induced by various imperfections and inconsistencies arising during fiber manufacturing. These include microscopic bends and variations in the diameter of the fiber, irregularities at the core–cladding boundary, and fluctuations in the refractive index distribution. 

In mode coupling, there is a transfer from low-order to high-order modes. Low-order modes correspond to the light propagation paths that pass through the center of the fiber core. They have smaller angles of incidence relative to the fiber axis and therefore suffer fewer reflections at the interface between the core and cladding, resulting in straighter and more direct paths along the fiber. They are more likely to be guided at the core for longer distances than higher-order modes [17].

The energy transfer to the higher-order modes is continuous and co-occurs with mode coupling [1] up to the SSD at the Zs point. When the light power distribution in the POF reaches an SSD, it maintains a constant angular distribution of the output light power, regardless of the angular distribution of the fiber entering it.

**Figure 1:**
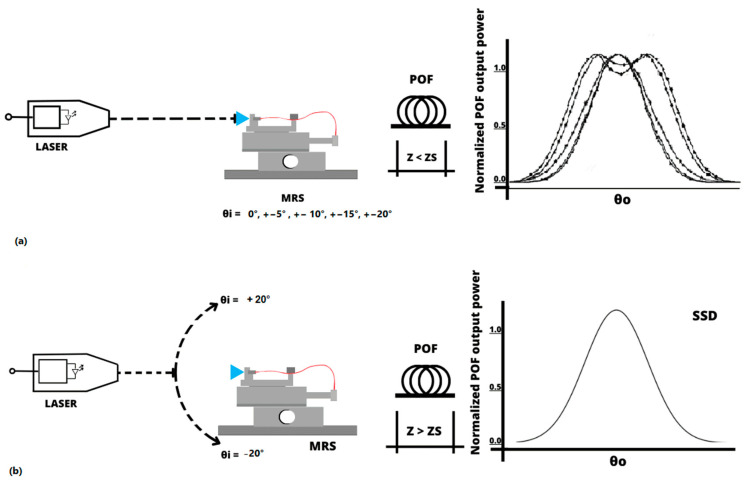
Output POF stretch for different conditions. MRS is Manual Rotation System goniometer, θi is light input POF angle, and θo is light output POF angle. (**a**) Angular light power distribution with POF length shorter than Zs length for θi varying inside interval −20° to +20°. (**b**) Angular output light power distribution with POF length longer than Zs for θi equal to any angle in interval −20° to +20°.

Figure 1 shows the POF angular light power distribution output when the light emitted by a laser is introduced into the fiber coupled to the manual rotation system (MRS) goniometer. Figure 1a illustrates that, for z < Zs, the distribution of angular light power in the POF changes depending on the angle at which light enters the POF (0°, ±5°, ±10°, ±15°, and ±20°). In this figure, each curve is for an input angle. Figure 1b, when z > Zs, shows that the angular light power distribution of the POF is independent of the angle at which the light enters the fiber.

This behavior indicates that for lengths z greater than Zs, the light of the POF reaches a state of mode stabilization, where the output light power distribution becomes the same for different injection angles.

Using the D-shape promotes the condition shown in Figure 1b, with shorter POF sections compared to without the D shape. For applications such as those mentioned in Section 1, using POFs (plastic optical fibers) of shorter lengths offers significant advantages. These advantages are clearly represented in Figure 1b, which shows that shorter POFs are easier to use and consume less space, thereby providing an efficient solution for embedded systems.

To solve the problem of modal dispersion, researchers have developed various techniques, which are listed in Table 1:

These methods often require adding external components or altering the fiber during manufacturing, potentially increasing volume, weight, and costs and impacting reliability.

## 3. Material and Methods, Components, and Experimental Setup

**Figure 2:**
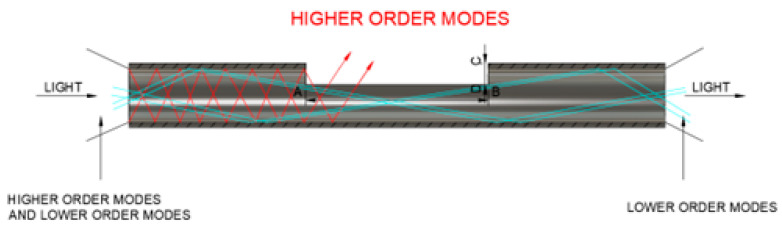
A schematic diagram of the D-shape in POF.

This work introduces a D-shaped design to filter out high-order modes effectively, as shown in Figure 2. The AB dimension represents the width of the D-shape of 20 mm, and the CD dimension represents its depth of 0.25 mm. The optical beam enters the POF, passes through the D-shaped region, and exits on the opposite side. Due to their steep propagation angles, high-order modes refract at the interface between the D-shaped core and surface. Meanwhile, low-order modes traverse the D-shaped area until they exit the fiber’s end.

For the evaluation of the behavior of modes along the POF, the power flow equation developed by Gloge was applied [26]. Based on the assumption that mode coupling in multimode optical fibers occurs between neighboring modes, Gloge formulated the time-independent power flow equation as follows:

(1) $$\frac{\partial P\left(\theta ,z\right)}{\partial z}=-\alpha \left(\theta \right)P\left(\theta ,z\right)+\frac{D}{\theta }\frac{\partial }{\partial \theta }\left(\theta \frac{\partial P\left(\theta ,z\right)}{\partial \theta }\right)\;$$

P(θ,z) is the angular light power distribution in the POF, D is the mode coupling coefficient, θ is the propagation angle in the POF core, and z is the reference point of the POF length relative to the light input.

To solve the differential equation proposed by Gloge [27], we adopted the implementation of numerical methods based on explicit finite differences. This approach allowed us to develop a robust and efficient computational solution to the equation. As a result of implementing these methods, we derived a reformulated version of Equation (1):

(2) $$P_{i,j+1}=\left(\frac{\Delta zD}{\Delta \theta^{2}}-\frac{\Delta zD}{2\theta i,j\Delta \theta }\right)P_{i-1j}+\left(1-\frac{2\Delta zD}{\Delta \theta^{2}}\right)P_{i,j}+\left(\frac{\Delta zD}{2\theta i,j\Delta \theta }+\frac{\Delta zD}{\Delta \theta^{2}}\right)P_{i+1j}\;$$

**Figure 3:**
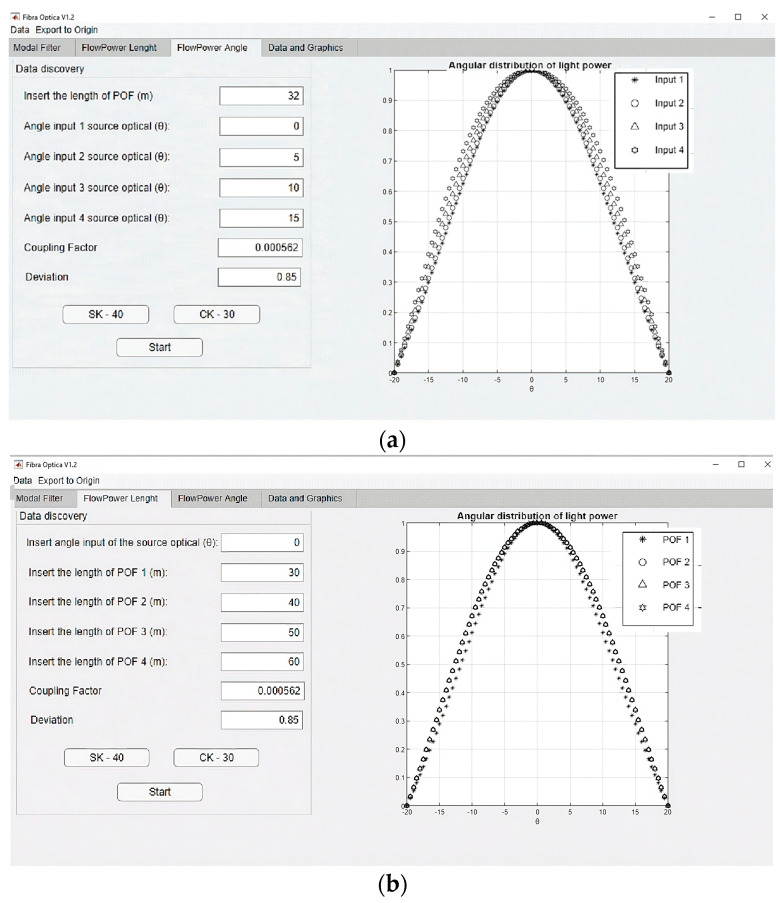
Screenshots of the application developed. (**a**) The simulation result with a single POF length and four different angles of light entering the POF; (**b**) simulation with a single angle of light entering the POF and four different POF lengths.

A dedicated computational tool was developed to apply the Gloge equation and investigate the behavior of the modes along the POF. The tool was created in MATLAB using the APP Designer, which enables the manual creation of a graphical user interface (GUI). As shown in Figure 1, this tool considers different angles of light entering the POF to analyze the angular light power distribution at the POF output from two perspectives: 1—the angle of light entering the POF; 2—the length of the POF. Figure 3 is a screenshot of the development of the application. It shows the angular light power distribution at the POF output, considering the idea in Figure 1.

Figure 3a shows that it is possible to see that there is a length of the POF from which the convergence of the angular light power distribution at the POF exit becomes evident. For the example in Figure 3a, this length is around 30 m. This length permits the investigation of light propagation at various light-entering angles in the POF. As one can see in Figure 3b, the output power distribution is almost constant for POF lengths longer than 30 m for several entering angles. Figure 3a,b shows that the POF lengths determine the mode stabilization for all input angle conditions.

This result was compared to experimental data, showing excellent concordance.

A preliminary step in conducting simulations that elucidate the modal behavior within the POF involves determining the mode coupling coefficient, denoted as “D”. This coefficient quantifies the rate of energy transfer between the various modes.

The method described in the literature was employed to calculate D [28]. In order to measure the variance in the light intensity at the outputs of the POF with lengths z1 and z2 and accurately calculate the coupling factor D as established in Equation (3), we implemented the methodology described below:

1. Collecting the light: The first step consists of collecting the light at the ends of the POFs with lengths z1 and z2, where z1 and z2 are lengths of the POF with z > 0 [25]. To capture the light at the exit of the POF, a CCD is positioned 2 mm from the end face of the POF.
2. Image processing: We then transferred the image obtained by the CCD to ImageJ 1.53r software. This platform allows for not only detailed visualization of the image but also precise extraction of the numerical data related to the angular light power distribution.
3. Statistical analysis in Origin: With the angular light power distribution curve data obtained by far-field imaging using ImageJ software, we proceeded to perform a statistical analysis using Origin 2019b software. This software was used to calculate the variance in the angular light power distribution for lengths z1 and z2 of the POF.

This method ensures the accuracy of the variance data obtained, which is confirmed by the literature [29,30,31,32]. These variance values are essential and are applied directly to Equation (3), providing a solid basis for determining the coupling factor. The integration of these advanced techniques and tools, from light collection to statistical analysis, establishes a reliable protocol for investigating the transmission characteristics of POFs.

(3) $$D=\frac{\sigma_{z_{2}-}^{2}\;\sigma_{z_{1}\;}^{2}}{2\left(z_{2}-z_{1}\right)}$$

To corroborate the experimentally determined and simulated Zs values, Equation (4), as defined in previous studies [30,31,32,33], was employed to ascertain the Zs length.

(4) $$Zs=\frac{0.2}{D}*{\left(\frac{NA}{n_{1}}\right)}^{2}\;$$

where D is the mode coupling coefficient, NA is the numerical aperture, and n_1_ is the refractive index of the core.

**Figure 4:**
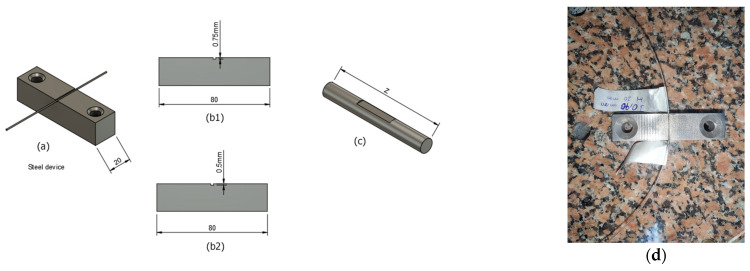
The device for creating the D-shape in POFs. (**a**) The D-shaped insertion device, (**b1**) a side view of the device for creating the D-shape in a 1 mm diameter POF, and (**b2**) a side view of the device for creating the D-shape in a 0.75 mm diameter POF. (**c**) A POF with a D-shape. (**d**) A POF accommodated in the steel device.

This study adopted the D-shape to induce SSD in shorter POF segments instead of incorporating additional components into the fiber. The process involved the development of a specialized device, which served as the basis for grinding the fiber body to achieve the desired D-shaped effect. Two devices with depths of 0.5 mm and 0.75 mm were fabricated. Figure 4a shows a steel device specially developed for grinding the POF, equipped with two supports that ensure the POF is securely fixed during the process. Figure 4(b1,b2) show, in detail, the cavities of this device, designed to precisely fit POFs with diameters of 1 mm and 0.75 mm, respectively. During manual sanding, the POF is accommodated in these cavities, leaving only the area to be sanded exposed. Figure 4d shows how the POF is positioned in the cavity prepared for sanding, while Figure 4c highlights the result of the POF after receiving the D-shaped finish.

POFs with 1 mm and 0.75 mm diameters were utilized in this study. Figure 4a illustrates the device used to grind the POF and create the D-shaped configuration.

When using the device in Figure 4(b1), there is a reduction in the POF diameter in the D-shaped region from 1 mm to 0.75 mm and from 0.75 mm to 0.50 mm with the device in Figure 4(b2), depending on the POF used. Figure 4c illustrates the D-shape after the sanding process. The length and depth of the groove determine the carachteristics of the D-shape in the POF. When analyzing the light losses of the polishing methods, it was found that using a lower grit sandpaper, which results in a less rough surface, reduced light scattering, increasing the amount of light transmitted through the D-shape. On the other hand, using rougher grits, such as P400, reduced the initial POF transmitted signal.

In summary, the key to optimal D-shaped filter operation lies in striking a perfect balance between surface roughness and transmission losses due to polishing. Analyses from prior research suggest that this equilibrium is attainable by employing P600 grit sandpaper for polishing the D-shape [14]. The ESKA SK40 POF, whose specifications are detailed in Table 2, was the fiber chosen for this study. This fiber was selected because of its exemplary performance as a commercial component designed for light transmission.

Upon evaluating the initial results, the decision was made to extend the application of the outlined methodology to another type of fiber, specifically the POF ESKA CK30, whose characteristics are presented in Table 3.

**Figure 5:**
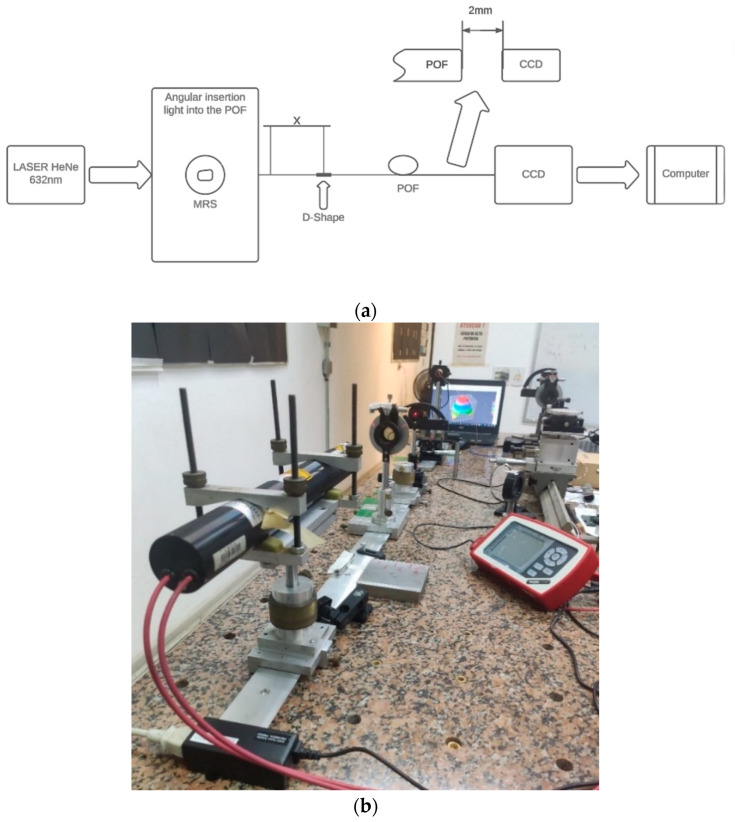
(**a**) A block diagram of the setup for measuring angular light power distribution using MRS, and (**b**) an image of the setup in the laboratory.

A helium–neon (He-Ne) laser at 632 nm was used as the light source, and a manual rotation stage (MRS) was used to control the angle of light injection into the POF, and at the output of the POF, a CCD connected to a computer registered an FFP by software was used. Figure 5a shows the block diagram of the setup, and Figure 5b shows the setup in the laboratory.

According to Figure 5, the He-Ne laser was positioned so its light could be coupled to the POF under evaluation. Using the MRS, the injection angles were selected as 0, 5, 10, 15, and 20 degrees.

The aim of varying the length of the fiber under test is to obtain a more comprehensive set of data that allows the distribution of angular light power and the state of SSD to be evaluated. As already mentioned, various POF z lengths were used for the evaluation.

## 4. Experimental Results

Determining the mode coupling coefficient, D, was the starting point for comparing the simulations and measurements. The variance in the CCD-detected optical signal beam distribution for various POF lengths was obtained to ascertain D. Utilizing the POF sections accessible in the laboratory, z-values of 5, 10, and 22 m were employed. The variance values were obtained with and without the D shape. Table 4 shows the variance values.

After determining the variances, the values were subsequently inserted into Equation (2) to derive the modal coupling coefficient, as shown in Table 5.

**Figure 6:**
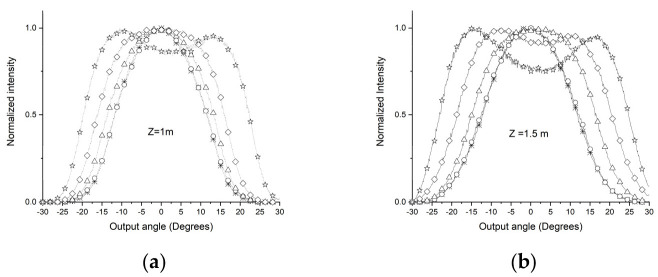

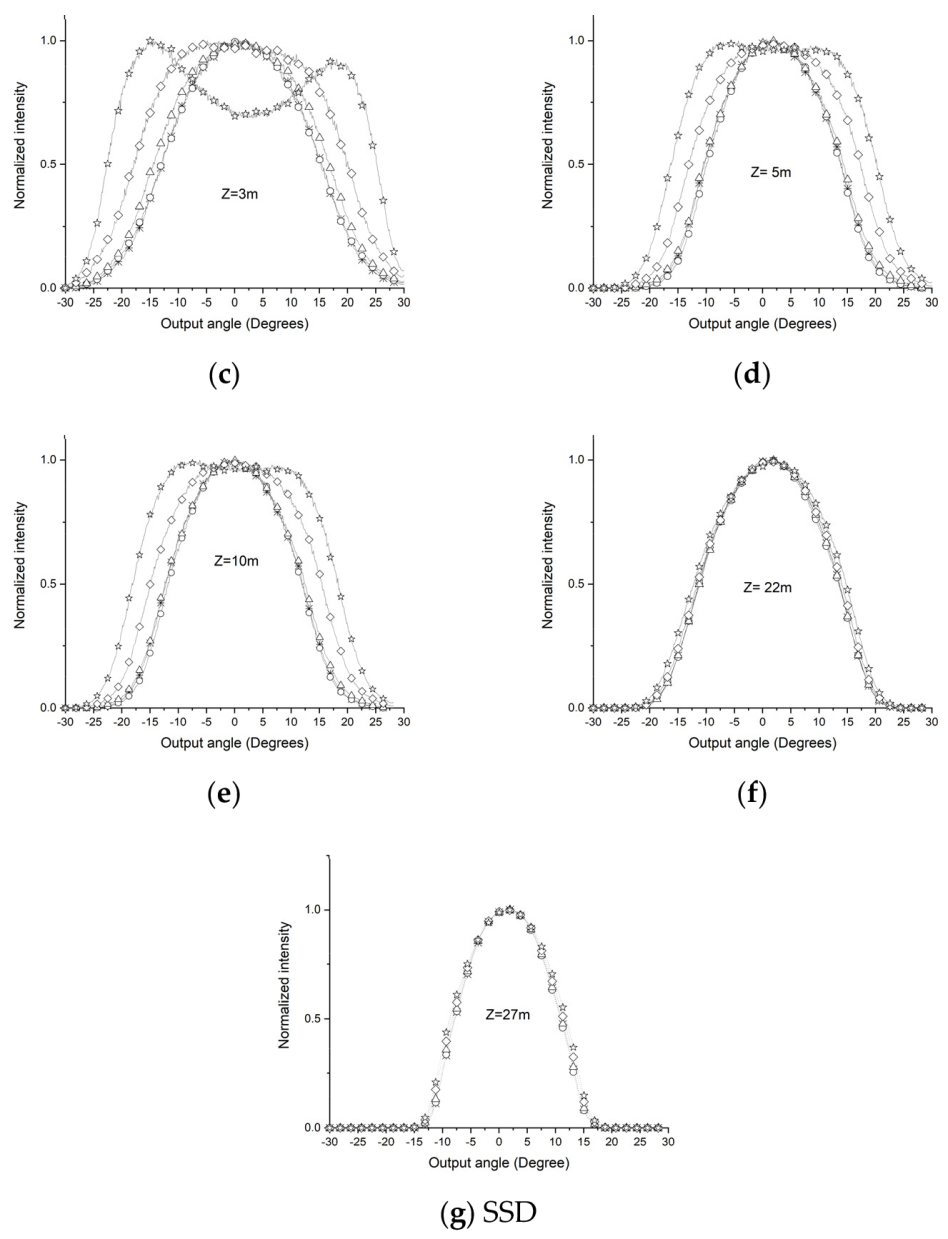
Output light power distribution for the ESKA SK40 POF for various input angles without using D-shape (☆ = +−20°, ◊ = +−15°, Δ = +−10°, ○ = +−5°, and * = 0°).

The values obtained for the D parameters are compatible with those of previous authors [7,25,28]. Figure 6a–g shows the experimental results of the output light power distribution of the POF ESKA SK40 without using the D shape for various input angles. The measurements were taken with 1, 1.5, 3, 5, 10, 22, and 27 m of POF stretches. Figure 6g shows the establishment of the SSD. This finding aligns with the simulations conducted through the tool developed and other previously published studies [25,28]. The angular distribution of light power is a function of the POF input angle for all stretches considered [28]. The occurrence of SSD, as depicted in Figure 6g, in which the angular light power distribution of the POF output remains constant, materializes exclusively when the fiber length (z) exceeds the threshold length (Zs) [30].

The series of figures from 6a to 6g clearly demonstrate how the angular light power distribution changes with the extension of the POF’s length (z), distinctly showing that the SSD is achieved at a length of 27 m, as specifically highlighted in Figure 6g.

**Figure 7:**
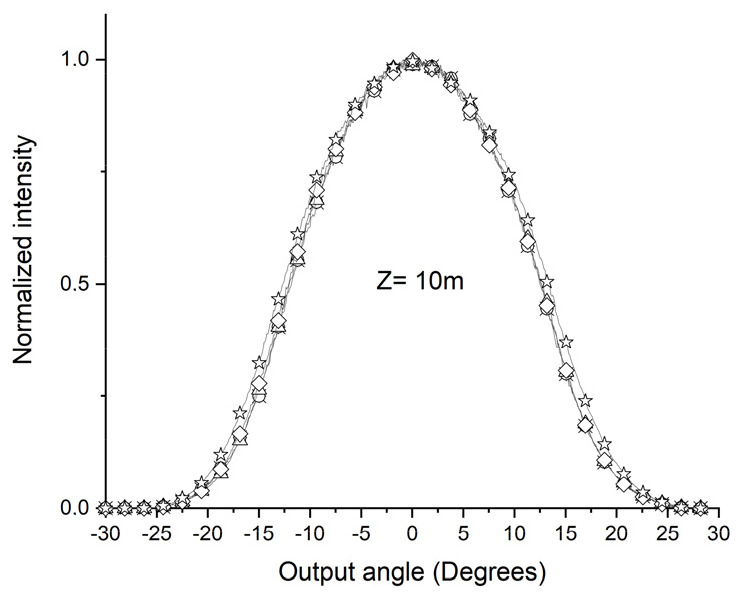
Output light power distribution for the ESKA SK40 POF for various input angles and z = 10 m with a D shape (☆ = +−20°, ◊ = +−15°, Δ = +−10°, ο = +−5°, and ∗ = 0°).

After taking the first measurements at the specified lengths, the D shape, always positioned at the same distance, x, from where the light is injected into the POF, was incorporated into the ESKA SK40 POF, as shown in Figure 5a. A 10 m section was applied using the device shown in Figure 4(b1) to insert the D shape into the POF. Figure 7 shows a change in the angular light power distribution compared to Figure 6e by inserting the D shape. The convergence of the angular light power distribution becomes visible, characterizing the SSD. The 10 m length obtained by the SSD when using the D shape aligns with the simulations and calculations of 10.4 m.

In the second part of the experiment, the ESKA CK30 POF, featuring the characteristics detailed in Table 3, was employed to implement the procedure on an alternative fiber. Stretches of 1, 3, 5, 10, 15, 20, and 30 m without a D shape were employed. Table 6 presents the variance in output light power distribution for the ESKA CK30 POF across different sections: 15 and 20 m without a D shape and 5 and 10 m with a D shape incorporated.

The variance data acquired were utilized in Equation (2) to derive the mode coupling coefficient values, as detailed in Table 7, which presents the coefficients for the POF ESKA CK30.

**Figure 8:**
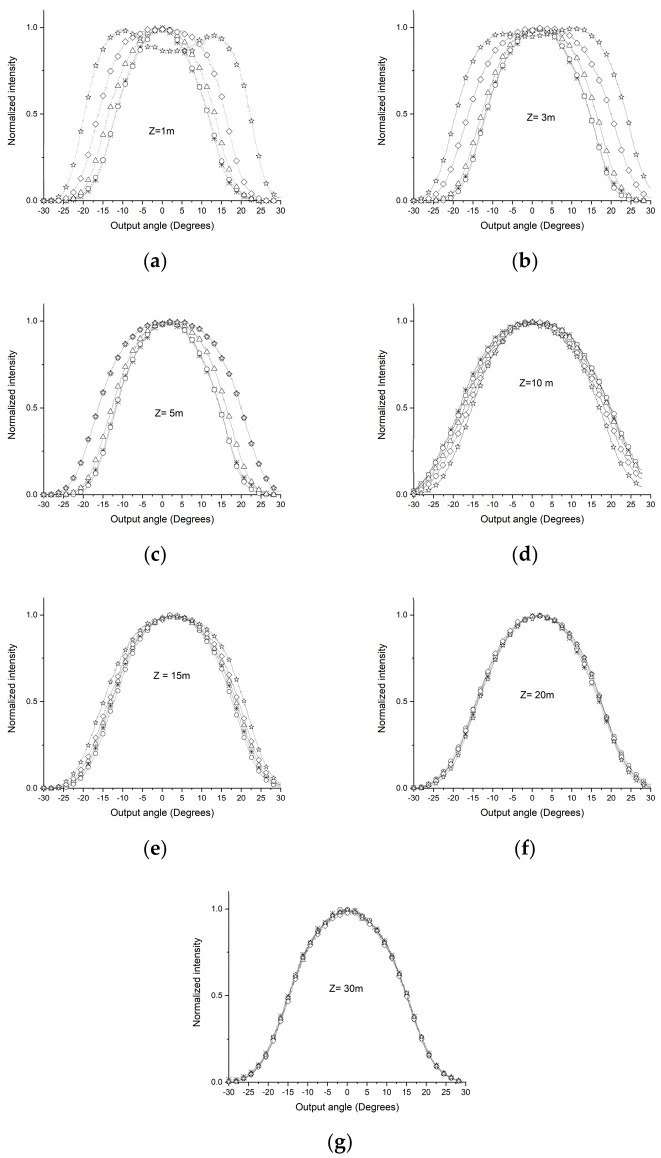
Output light power distribution of the ESKA CK30 POF for various POF input angles and z lengths, obtained experimentally without using D-shape (☆ = +−20°, ◊ = +−15°, Δ = +−10°, ο = +−5°, and ∗ = 0°).

Figure 8 illustrates the resultant light power distribution across distinct segments of the POF. By comparing the theoretical and experimental Zs for the CK-30 POF, one can see a different condition from the SK-40. The CK-30 is a higher attenuation POF and will be used to validate the presented analysis.

The series of figures from Figure 8a–g clearly demonstrate how the angular light power distribution changes with the extension of the POF’s length (z), distinctly showing that the SSD is achieved at a length of 20 m, as specifically highlighted in Figure 8f.

After acquiring angular light power distribution patterns within different segments of the POF up to the identification of the SSD, the subsequent step involved implementing the D-shape process in particular segments where the absence of SSD had been observed in the previous measurements conducted without the D-shape process. The dimensions of the device to create a D shape are shown in Figure 4(b2). Using the data from the output light power distribution captured by the CCD, the variance in the optical beam injected into two sections of the POF was determined, following the same procedure as previously. As can be seen, the SSD was not obtained with a 10 m stretch of the POF in Figure 8d; after the insertion of the D shape, the SSD was observed.

**Figure 9:**
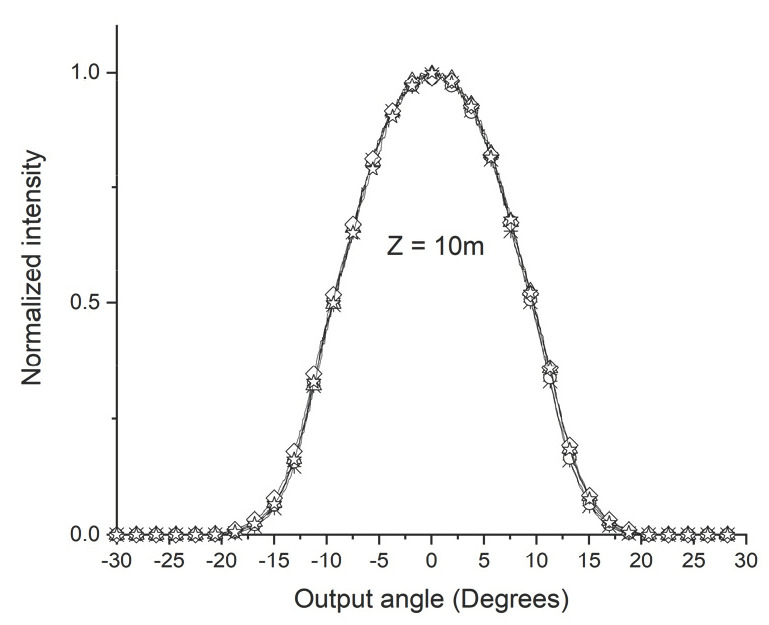
Output light power distribution for the ESKA CK30 POF for various input angles and z = 10 m using the D shape, obtained experimentally (☆ = +−20°, ◊ = +−15°, Δ = +−10°, ο = +−5°, and ∗ = 0°).

**Figure 10:**
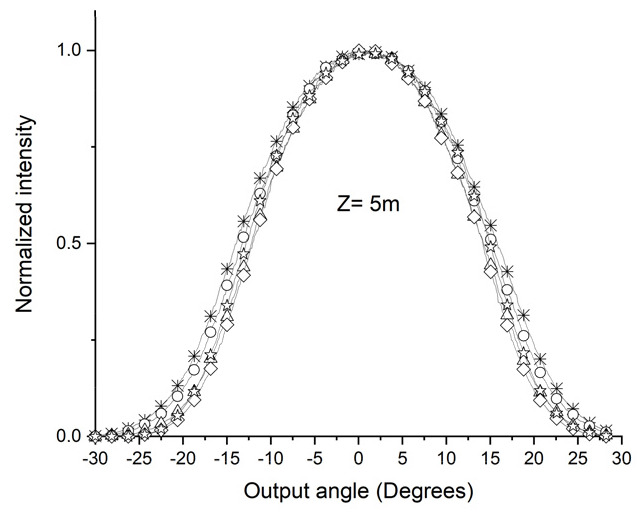
Normalized output light power distribution for the ESKA CK30 POF for various input angles and z = 5 m using the D shape, obtained experimentally (☆ = +−20°, ◊ = +−15°, Δ = +−10°, ο = +−5°, and ∗ = 0°).

Figure 9 shows the CK30 POF with a 10 m stretch using the D-shape, showing complete convergence of the angular light power distribution for different entry angles into the POF. In contrast, in Figure 10, with the same POF with a 5 m stretch, we observe that convergence of the angular light power distribution does not occur ultimately, which indicates that the value of Zs is greater than 5 m and less than 10 m.

The calculated Zs value for the ESKA CK30 POF with the D shaped insert was 7.56 m. Experimentation using a 5 m long POF was carried out to validate the agreement with the experimental results. Figure 10 shows the modal behavior of the ESKA CK30 POF with the D shape. The 5 m section did not exhibit complete convergence of the output light power distribution, even after introducing the D shape, aligning with the previously calculated Zs value.

From Figure 9 and Figure 10, it is possible to conclude that SSD is between 5 and 10 m.

Table 8 shows a comparative analysis of the reduction in Zs obtained by applying the D-shape technique to the POF. Can we say that the D-shape technique significantly reduces the Zs value, thereby eliminating the need to incorporate supplementary elements, intentional curvatures, or bending deformation into the POF normally used for this task? On the other hand, when using the technique known as Strained on the POF [23], a considerable reduction in the Zs value is also observed. However, it is essential to note that this approach requires a significant area of 300 mm × 200 mm and twelve 10 mm diameter cylinders to induce bending deformations in the POF and achieve SSD.

The Zs values obtained experimentally were compared with the calculated values. It can be seen that in the reference experiment [25], there is an 88% divergence between the experimental value of Zs (49 m) and the calculated value (26 m) without using the Strain technique. When the Strain technique is applied, the difference between the experimental value (2.5 m) and the calculated value (1.46 m) drops to 71%. In this work, the experimental Zs value for POF ESKA SK40 without the application of the D-shape technique was 27 m, and the calculated value was 25.94 m, a difference of 4%; the experimental Zs value for POF ESKA CK30 without the D shape was 20 m, and the calculated value was 19.3 m, a difference of 3.6%.

## 5. Discussion and Bit Error Rate (BER) Test

**Figure 11:**
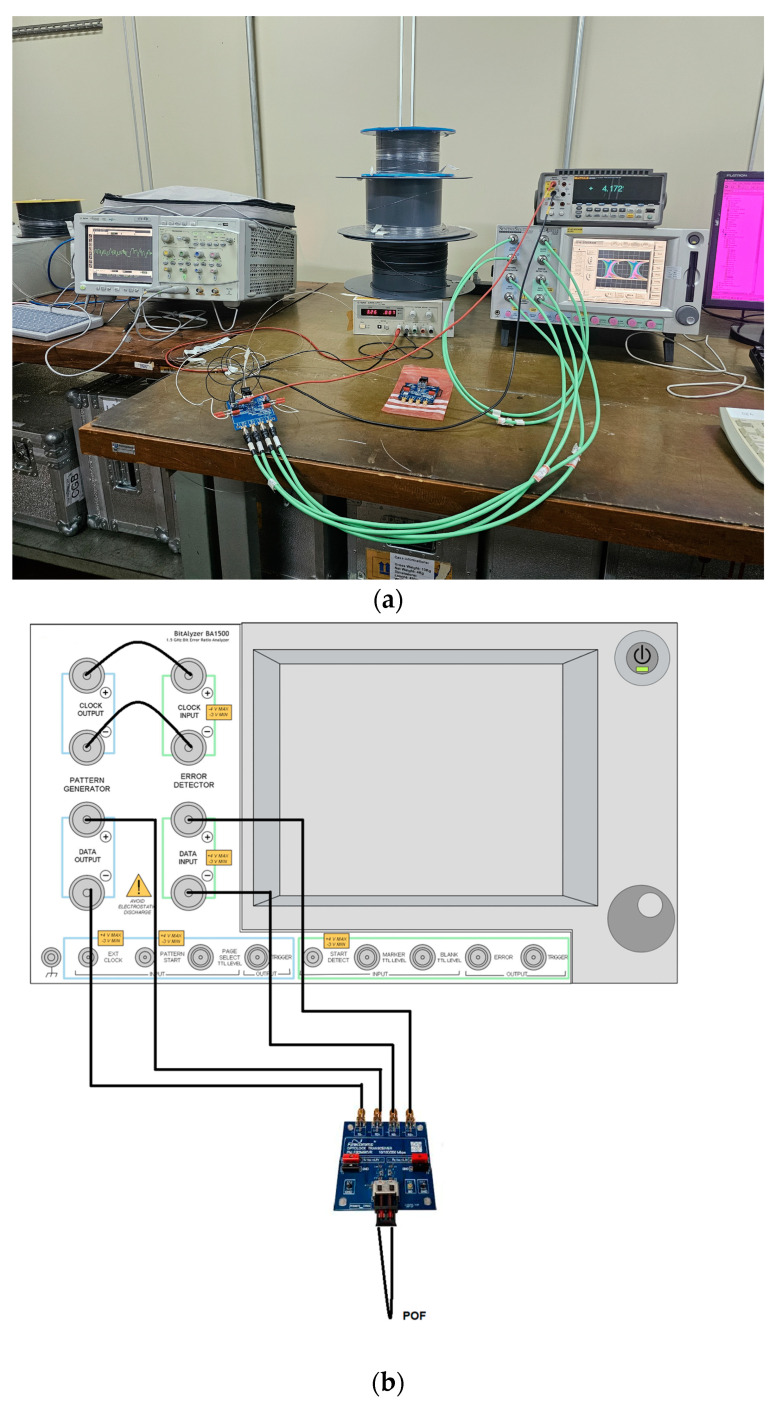
(**a**) Image of setup for BER evaluation. (**b**) Ber analyzer and Firecomms evaluation connection diagram.

As mentioned in Section 1, tests were carried out on POF optical links to assess the effects of inserting or not inserting the D shape. The bit error rate (BER) values were obtained using the BER analyzer model BA 1500 Synthesis and the Firecomms evaluation boards, specifically the FB2M5KRR, with transmission rates of up to 250 Mbps. A 95% confidence level was chosen as the threshold for a specified bit error rate (BER) of 1.10^−9^. This same confidence level is used to estimate the BER through statistical methods [34]. Thus, for a bit rate of 250 Mbps, the accumulation time was 12 s. Figure 11 shows the setup for the measurements.

The BA 1500 BER analyzer has its clock connections, clock output, and clock input; the test data generated according to the chosen configuration (PRBS-7, Burst minimum length 32, and integration period 1 × 10^9^ bits) are sent through the outputs (Data Output) to the Firecomms FB2M5KRR evaluation board, where the data are converted from the electrical to the optical domain, passing through the POF and returning to the Data Input inputs.

Table 9 shows the BER for several different POF lengths. To experimentally evaluate the D-shaped applications, the CK-30 POF was chosen due to its higher attenuation coefficient, which represents the worst propagation conditions. The measurements presented are for POFs with and without a D shape. The evaluation board works with 650 nm. The obtained results enable the discussion of the D-shaped effect in the context of high-performing embedded applications.

From Table 9, one can see the mode equilibrium effect on light POF propagation. The BER values, without a D shape, increase as the optical fiber length increases, decrease as the length approaches Zs, and begin to increase again after Zs. For the CK-30 POF, the Zs equals 19.3 m, as shown in Table 7.

With a D shape, the BER values reveal the effects of shortening the Zs length due to the D shape introduction. First, all of the BER values are smaller than the values obtained without a D shape. Second, the BER values increase as the optical fiber length increases and decrease around the new Zs values as before. The BER increases as expected after the new Zs value of 10 m, since the effects of the dispersion mode arise with the optical fiber length.

For the shortest length in Table 8, despite a low value of the BER, resulting in reduced mode dispersion effects and attenuation, the D shape reduces it to a lower value.

**Figure 12:**
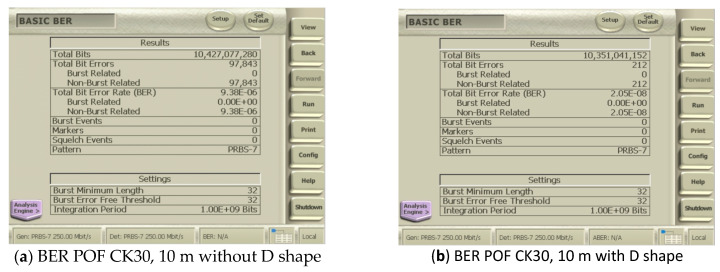
Comparative Evaluation of Bit Error Rate (BER) at a 250 Mbps Transmission Rate over a 10-m CK30 POF. (**a**) depicts the BER measurement without the D-shape insert, while (**b**) demonstrates the BER measurement with the D-shape insert, highlighting the impact of the insert on transmission quality.

Figure 12 shows the screen of the BER BA 1500 analyzer when evaluating the POF CK30 at 10 m with and without the D format. We observed the following values: 9.38 × 10^−6^ without the D format in Figure 12a and 2.05 × 10^−8^ with the D shape in Figure 12b.

If we consider the lengths of the POF to be beyond the Zs length, we note in our experiments that the D shape still improves the bit error rate (BER). In a 30 m segment, we observed an improvement in the BER from 1.03 × 10^−6^ to 4.83 × 10^−7^.

## 6. Conclusions

This study investigated the characteristics of the output light power distribution of the step-index POF, with and without a D shape. Optical propagation analysis along the POF was simulated and conducted experimentally. The simulation analysis was based on the Gloge equation solution using a simulation tool developed by the authors. The experimental analysis used ESKA SK40 POF with a diameter of 1 mm. The optical propagation analysis results were applied to a general POF, such as the ESKA CK30 (0.75 mm diameter), which had an excellent agreement for simulation and measured data. The results confirm that the optical propagation analysis conducted is robust. A modal coupling investigation was conducted, which considered the D shaped fiber as a solution to filter high-order modes without the need for additional components or more complex setups.

These findings suggest that the D shape effectively reduces the mode stabilization length and, by extension, minimizes mode dispersion effects. Such a reduction facilitates more efficient data transmission in embedded systems that employ POFs as an internal data bus medium. This advance in understanding the properties of D shape-modified POFs paves the way for significant improvements in optical communication systems’ reliability and performance, particularly in applications where signal integrity and reliability are critical, as mentioned in Section 1, where the involved lengths are short.

This study also opens up the possibility of using POFs with a D shape in different devices to replace short-distance electrical connections, such as computers, robots, and others.

## Figures and Tables

**Table 1:** Techniques and methods used to manage and control modal dispersion in optical fibers.

Technique	Method	Reference
Mode Conditioning	To eliminate high-order modes, an element such as a lens or a specialized diffuser is used at the beginning of the fiber to control the way the light enters.	[18]
Offset Launch Technique	It involves coupling light off-center relative to the fiber’s axis, helping to reduce modal dispersion.	[19]
Modal Filters	These filters, attached to the fiber’s leading edge, allow low-order modes to pass while blocking high-order ones.	[20]
Narrow Spectral Width Sources	Light sources with narrow spectra and high power can minimize power transfer between modes.	[21]
Twist Processing for Mode Control	This method modulates modal energy distribution, encouraging energy transfer from lower to higher-order modes by twisting the fibers.	[22]
Refractive Index Profiling	Adjusting the fiber core’s refractive index during production can reduce high-order mode excitation.	[23]
Mode Scrambler	This technique aims to create controlled disturbances in the propagation modes of light within a fiber. It does this by using specially made cylinders that bend the Plastic Optical Fiber (POF). This bending process facilitates the coupling or interaction between different modes of light within the fiber, enhancing the fiber's performance for specific applications.	[24]
Airgap Filter	A spatial filter creates an air gap, reducing the numerical aperture and modal dispersion.	[17]
Microstructured POF	Designing the fiber with a specific pattern of air holes reduces the number of propagation modes, minimizing modal dispersion.	[7]
Strained POF	Applying strain to the fiber enhances mode coupling, reducing dispersion.	[25]
Mandrel Wrap	This consists of wrapping a section of optical fiber around a cylindrical mandrel of a specified diameter for several turns. This creates a bend that induces additional losses for higher-order modes without significantly affecting lower-order modes.	[26]

**Table 2:** Features of POF ESKA SK40.

Core material	Polymethyl–Methacrylate Resin
Cladding material	Fluorinated Polymer
Core refractive index	1.49
Refractive index profile	Step Index
Numerical Aperture	0.5
Core diameter	980 μm
Cladding diameter	1000 μm (1 mm)
Transmission loss at 650 nm	150 dB/km

**Table 3:** Features of POF ESKA CK30.

Core material	Polymethyl–Methacrylate Resin
Cladding material	Fluorinated Polymer
Core refractive index	1.49
Refractive index profile	Step Index
Numerical Aperture	0.5
Core diameter	735 μm
Cladding diameter	750 μ (0.75 mm)
Transmission loss at 650 nm	200 dB/km

**Table 4:** The variance in the launch beam distribution σ2 for POF ESKA SK40, with and without the D shape.

	Stretch of the POF under Investigation	The Variance in the Launch Beam Distribution σ²
POF without D-shape	Z1 = 5 m	$\sigma_{1}^{2}$ = 0.11328
	Z2 = 22 m	$\sigma_{2}^{2}$ = 0.14279
POF with D-Shape	Z1 = 5 m	$\sigma_{1}^{2}$ = 0.15354
	Z2 = 10 m	$\sigma_{2}^{2}$ = 0.13203

**Table 5:** Mode coupling coefficient, D, values with and without D-shaped applications in POF ESKA SK40.

Mode coupling coefficient without the use of a D shape	8.6794 × 10^−4^ (rad^2^/m)
Mode coupling coefficient with the use of a D shape	2.151 × 10^−3^ (rad^2^/m)

**Table 6:** The variance in the launch beam distribution, σ_2,_ for POF ESKA CK30, with and without a D shape.

	Stretch of the POF under Investigation	The Variance in the Launch Beam Distribution σ2
POF without D shape	Z_1_ = 15 m	$\sigma_{1}^{2}$ = 0.14397
	Z_2_ = 20 m	$\sigma_{2}^{2}$ = 0.13229
POF with D Shape	Z_1_ = 5 m	$\sigma_{1}^{2}$ = 0.1197
	Z_2_ = 10 m	$\sigma_{2}^{2}$ = 0.14946

**Table 7:** Mode coupling coefficient, D, values with and without D-shaped application in POF ESKA CK30.

Mode coupling coefficient without the use of a D shape	1.168 × 10^−3^ (rad^2^/m)
Mode coupling coefficient with the use of a D shape	2.976 × 10^−3^ (rad^2^/m)

**Table 8:** Comparative analysis of Zs reduction utilizing D shape and Strained techniques.

	POF SI TORAY: PFU-CD1001-22E (REF [23])	POF SI ESKA SK40 MITSUBISHI RAYON (This Work)	POF SI ESKA CK30 MITSUBISHI RAYON (This Work)
The refractive index of the core (n0)	1.492	1.49	1.49
Numerical aperture (NA)	0.46	0.5	0.5
Transmission loss	150 dB/Km @ 650 nm	150 dB/Km @ 650 nm	200 dB/Km @ 650 nm
Mode coupling coefficient, D (rad²/m)	Unstrained 7.3 × 10^−4^	Without D shape 8.6794 × 10^−4^	Without D shape 1.191 × 10^−3^
	Strained 1.3 × 10^−2^	With D shape 2.151 × 10^−3^	With D shape 2.976 × 10^−3^
Mode Stabilization Length, Zs (m)	Unstrained 49 (experimental) 26 (calculated)	Without D shape—27 (experimental) 25.94 (calculated)	Without D shape 20 (experimental) 19.3 (calculated)
	Strained 2.5 (experimental) 1.46 (calculated)	With D shape 10 m (experimental) 10.4 (calculated)	With D shape 5~10 (experimental) 7.57 (calculated)
Core diameter (mm)	1000 μm	980 μm	735 μm

**Table 9:** Comparative analysis of BER with and without the insertion of the format D in the POF.

Length	BER without D Shape	BER with D Shape	Ratio
1	7.6 × 10^−7^	3.7 × 10^−10^	2054
3	4.57 × 10^−7^	6.8 × 10^−8^	6.7
10	9.38 × 10^−6^	2.0 × 10^−8^	469
20	1.2 × 10^−7^	8.8 × 10^−8^	1.36
30	1.03 × 10^−6^	4.8 × 10^−7^	2.5

## Data Availability

The raw data supporting the conclusions of this article will be made available by the authors upon request.
